# Monthly Notes

**Published:** 1890-01

**Authors:** 


### Monthly Notes.

Mr. H. E. Smith, of G. F. Harvey & Co., manfacturing chemists, of Chattanooga, Tennessee, called and showed us some of their celebrated preparations, and stated that he was doing very well here. The above firm have just moved from Saratoga, New York, so that they are one of us now.

Dr. H. M. Pinkard has been in Atlanta calling on our physicians and leaving specimens of Wm. R. Warner & Co.'s preparations, and all enjoyed his good nature and ready wit.

